# Positive correlation between wood *δ* ^15^N and stream nitrate concentrations in two temperate deciduous forests

**DOI:** 10.1088/2515-7620/ab77f8

**Published:** 2020-02-27

**Authors:** Robert D Sabo, Andrew J Elmore, David M Nelson, Christopher M Clark, Thomas Fisher, Keith N Eshleman

**Affiliations:** 1 Oak Ridge Institute for Science and Education, United States Environmental Protection Agency, Office of Research and Development, National Center for Environmental Assessment, US EPA (8623-P),1200 Pennsylvania Ave NW; Washington, DC 20460, United States of America; 2 University of Maryland Center for Environmental Science, Appalachian Laboratory, 301 Braddock Road, Frostburg, MD 21532, United States of America; 3 United States Environmental Protection Agency, Office of Research and Development, National Center for Environmental Assessment, US EPA (8623-P),1200 Pennsylvania Ave NW; Washington, DC 20460, United States of America

**Keywords:** nitrogen, nitrate, stable isotopes, forest, stream, oligotrophication

## Abstract

A limitation to understanding drivers of long-term trends in terrestrial nitrogen (N) availability in forests and its subsequent influence on stream nitrate export is a general lack of integrated analyses using long-term data on terrestrial and aquatic N cycling at comparable spatial scales. Here we analyze relationships between stream nitrate concentrations and wood *δ*^15^N records (n = 96 trees) across five neighboring headwater catchments in the Blue Ridge physiographic province and within a single catchment in the Appalachian Plateau physiographic province in the eastern United States. Climatic, acidic deposition, and forest disturbance datasets were developed to elucidate the influence of these factors on terrestrial N availability through time. We hypothesized that spatial and temporal variation of terrestrial N availability, for which tree-ring *δ*^15^N records serve as a proxy, affects the variation of stream nitrate concentration across space and time. Across space at the Blue Ridge study sites, stream nitrate concentration increased linearly with increasing catchment mean wood *δ*^15^N. Over time, stream nitrate concentrations decreased with decreasing wood *δ*^15^N in five of the six catchments. Wood *δ*^15^N showed a significant negative relationship with disturbance and acidic deposition. Disturbance likely exacerbated N limitation by inducing nitrate leaching and ultimately enhancing vegetative uptake. As observed elsewhere, lower rates of acidic deposition and subsequent deacidification of soils may increase terrestrial N availability. Despite the ephemeral modifications of terrestrial N availability by these two drivers and climate, long-term declines in terrestrial N availability were robust and have likely driven much of the declines in stream nitrate concentration throughout the central Appalachians.

## Introduction

Recent changes in climate, atmospheric chemistry, and disturbance have the potential to influence the productivity and export of nutrients from forested catchments by either constraining or enhancing nitrogen (N) availability [Elmore *et al* 2016, McLauchlan *et al* 2017, Peñuelas *et al* 2017]. Terrestrial N availability may increase from warming soils further stimulating mineralization, atmospheric N deposition adding N to soils, or changes in microbial communities increasing mineralization rates as acidified soils recover from decades of elevated acidic deposition [Aber *et al* 1989, Rustad *et al* 2001, Sinsabaugh *et al* 2004, Sinsabaugh 2010, Brookshire *et al* 2011, Oulehle *et al* 2011, Lawrence *et al* 2015, Oulehle *et al* 2017, Lawrence *et al* 2019]. On the other hand, greater N demand resulting from factors such as longer growing seasons or elevated atmospheric CO_2_ concentrations could reduce N availability [Ollinger *et al* 2002, Norby *et al* 2016]. As inferred from foliar and wood chemistry and net mineralization and nitrification rate measurements in North American forests, the latter is supported by evidence that N availability has declined in temperate forests during the past three decades [McLauchlan *et al* 2010, Durán *et al* 2016, Elmore *et al* 2016, McLauchlan *et al* 2017, Groffman *et al* 2018]. Stream nitrate export has also declined since the mid-1990s throughout many forests of the eastern United States [Eshleman *et al* 2013], which could result from declines in terrestrial N availability [Elmore *et al* 2016]. However, few studies have co-located records of terrestrial N availability and stream water chemistry with which to assess this hypothesis.

A general lack of paired analysis of terrestrial and aquatic N cycling at comparable spatial scales limits understanding of the drivers of long-term trends in terrestrial N availability and its subsequent influence on stream nitrate export. Stream nutrient data are often only obtained for limited periods or measured only periodically [Argerich *et al* 2013, Durán *et al* 2016], and such snapshots may not be adequate for comparison with longer-term indicators of terrestrial N cycling, such as deduced from tree-ring *δ*^15^N records [Gerhart and McLauchlan 2014]. While stream water nutrient data are generally considered integrative of an entire watershed [Likens 2017], in-stream processing could lead to an incongruity between stream and terrestrial N datasets [Scanlon *et al* 2010]. Tree-ring *δ*^15^N records have been suggested to be an integrated metric of soil N supply relative to plant demand [McLauchlan *et al* 2007, Howard and McLauchlan 2015, Sabo *et al* 2016a, McLauchlan *et al* 2017]. Wood *δ*^15^N integrates the effects of multiple fractionation pathways on N isotopes as it is transformed and lost in the ecosystem (e.g. nitrification, denitrification, etc). Overall, sites that have greater N availability tend to have higher rates of nitrification, denitrification, and leaching [Aber *et al* 1989, Galloway *et al* 2003, Pardo *et al* 2006, Pardo *et al* 2007, Templer *et al* 2007]. Greater N loss rates and fractionation leads to more ^14^N being lost from the system thus resulting in more isotopically positive *δ*^15^N signal remaining in the soil inorganic nitrogen (IN) pool available to plants [Sabo *et al* 2016a]. Overall, it has been hypothesized that stream nitrate and tree-ring *δ*^15^N should be positively correlated through time [McLauchlan *et al* 2007, Burnham *et al* 2016, Sabo *et al* 2016a], since stream nitrate concentrations are also thought to index terrestrial N availability [Aber *et al* 1989]. Long-term records of terrestrial N availability preserved in tree-ring *δ*^15^N data are typically normalized to the mean *δ*^15^N value of tree-ring time series to focus on temporal patterns [McLauchlan *et al* 2007], but doing so masks potential information about spatial variation in terrestrial N availability contained in non-normalized *δ*^15^N values and its relationship with stream nitrate [LeDuc *et al* 2013].

Catchments where long-term records of stream nutrient export exist and multiple tree-ring *δ*^15^N records of multiple species can be obtained represent promising locations for assessing the relationship between stream nitrate export and terrestrial N availability. For example, Sabo *et al* (2016b) observed higher mean (non-normalized) *δ*^15^N tree-ring values across a catchment in the Adirondack Mountains with higher mean annual flow-weighted nitrate concentrations relative to an adjacent catchment with lower nitrate concentrations (n = 20 tree-ring *δ*^15^N records per catchment), as well as declines in *δ*^15^N values of tree rings and stream nitrate concentrations for one of two subcatchments. However, a limitation of that study was that the water chemistry data spanned a period of only 11 years and that the most important tree species in one of the study catchments (*Fagus grandifolia*; American beech) could not be cored because it suffered from extensive disease and mortality [Sabo *et al* 2016a]. Thus, uncertainty remains about whether tree-ring *δ*^15^N records are representative of the relationship between terrestrial N availability and stream N export [Sabo *et al* 2016a].

To assess the relationship between terrestrial N availability and stream N export and to identify candidate drivers of the temporal variability of wood *δ*^15^N, we assembled dendroisotopic records for 96 trees and stream nitrate records for six small forested catchments in the central Appalachian Mountains. Rather than restrict our efforts to describing the temporal variability of *δ*^15^N for specific tree species, which ultimately may not be representative of catchment-wide changes in terrestrial N availability [Burnham *et al* 2016], we used a randomized sampling design to capture catchment-wide changes in wood *δ*^15^N. Furthermore, we used absolute non-normalized *δ*^15^N values to explore the spatiotemporal variation in terrestrial N availability and its relationship with stream nitrate concentration. Our objective was to test the hypothesis that spatial and temporal variation in terrestrial N availability observed in tree-ring *δ*^15^N records is associated with the spatial and temporal variation of stream nitrate concentration, respectively. To elucidate some of the potential drivers of terrestrial N availability, relationships among acidic deposition (nitrogen (N) + sulfur (S) deposition), precipitation, temperature, and forest disturbance on wood *δ*^15^N were also assessed.

## Methods

### Site description

**Figure 1.**
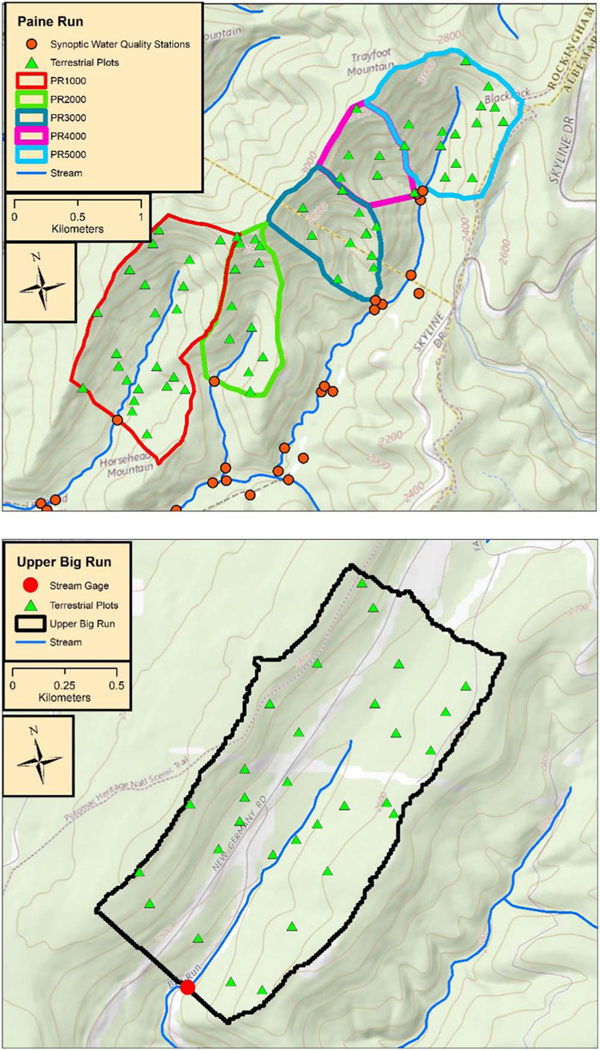
Maps of Upper Big Run and the five headwater catchments of Paine Run subjected to tree coring. The five headwater catchments were labeled PR1000, PR2000, PR3000, PR4000, and PR5000 from west to east since they contain unnamed tributaries, and these site IDs corresponded with individual trees sampled in the respective catchments.

The Upper Big Run catchment (UBR, 1.63 km^2^) is located within the Appalachian Plateau physiographic province in the western panhandle of Maryland, USA (figure 1, supplemental figure 1 is available online at stacks.iop.org/ERC/2/025003/mmedia). The underlying geology within the watershed consists of folded sedimentary rocks of Devonian through Mississippian age [Eshleman *et al* 1998]. Soils (Ultisols and Inceptisols) are primarily composed of stony loams of the Dekalb/Gilpin and Meckesville series [NRCS 2009]. According to the National Vegetation Classification System, the two primary ecological forest systems that occur within the catchment are Northeastern Interior Dry-Mesic Oak Forest and Appalachian Hemlock-Hardwood Forest [GAP 2011]. The watershed is 91% forested with the remainder consisting of meadows, roads, cropland, and a power-line right-of-way. Various silvicultural activities have been carried out in Upper Big Run since the 1970s and multiple insect defoliation events have been observed in the late-1980’s and mid-2000’s (supplemental figure 1; [Eshleman *et al* 1998, Townsend *et al* 2012].

The Paine Run catchment (PR, 12.7 km^2^) is located in the Blue Ridge in Shenandoah National Park, Virginia (figure 1, supplemental figure 1). The watershed falls within a designated wilderness area. Surficial geology of Paine Run is composed of phyllite, quartzite, and metasandstone. Soils (Ultisols and Entisols) are primarily composed of channery or stony loams of the Hartleton or Drall soil series. Numerous rubble islands are scattered throughout the catchment [NRCS 2009]. Paine Run is composed of secondary growth forests that have not been logged since the early 20th century. Vegetation surveys from the 1980’s show that chestnut oak (*Quercus prinus*) and various species of pine (*Pinus* spp.) were the dominant species. Widespread oak mortality in the early 1990’s was associated with repeated gypsy moth defoliation in the upper elevations of the watershed [Scanlon *et al* 2010]. Today, common forest types that occur within the catchment are Southern and Central Appalachian Oak Forest and Cove Forest along with scattered North-Central Appalachian Circumneutral Cliff and Talus systems [GAP 2011]. The five headwater catchments of Paine Run subjected to study each contain unnamed tributaries to the mainstem. As such, the catchments were labeled PR1000, PR2000, PR3000, PR4000, and PR5000 from west to east. These labels corresponded to the individual trees sampled in the respective catchments (supplemental table 5).

### Dendroisotopic records

Thirty trees at UBR and 66 trees at PR were cored at randomly located plots (figure 1). In the field, 706.86 m^2^ circular plots were established at the sampling points at UBR, and 225 m^2^ square plots were established within the five PR subcatchments (e.g., PR1000, PR2000, etc). Tree species composition surveys were completed during the fall and winter of 2014; all stems >5 cm diameter at breast height (dbh) within each plot were measured and identified. The bole of one individual of the species with the highest summed dbh in each plot was cored twice using a 5.15 mm incremental borer. In total, 18 different species were sampled at the two sites (supplemental table 5). The cores were returned to the lab, dried in an oven at 60 °C, sanded, and stored until ring widths were identified using CooRecorder software [Larsson 2009]. Two to three-year increments were generally cut from one bore per tree using a razor blade and stored in 96-well plates prior to *δ*^15^N analysis. Rings were cut along the grain in slices to ensure weighted sampling across years, thus representing a true weighted-average across each increment.

Approximately 10 mg of wood from the two to three-year increments for the 1980–2013 period was subsampled and used for *δ*^15^N and %N analysis. The midpoint of the aggregated range (e.g., 2003) was identified, and the preceding even year increment assigned. Results falling within 1986 to 2010 period were reported. Following the exact procedures described in Sabo *et al* (2016b), prepared samples (n = 1322) were analyzed for *δ*^15^N using a Carlo Erba NC2500 elemental analyzer (CE Instruments, Milano, Italy) interfaced with a Thermo Delta V+ isotope ratio mass spectrometer (Bremen, Germany). Following combustion in an elemental analyzer, a Carbosorb trap was used to remove CO_2_ and a magnesium perchlorate trap was used to remove water vapor. The *δ*^15^N data were normalized to the air reference standard scale using a two-point normalization curve with internal standards calibrated against USGS40 and USGS41 [Qi *et al* 2003, Brand *et al* 2014]. The analytical precision among runs (1*σ*) of an internal wood standard was 0.3‰. Catchment-scale tree ring *δ*^15^N values were estimated by taking the arithmetic mean of all tree ring *δ*^15^N values in a given time period for all trees within a watershed [Sabo *et al* 2016a].

### Atmospheric deposition, water quality, disturbance, and climatic data

Stream chemistry and discharge have been monitored at the outlet of UBR and PR since 1990 and 1992, respectively [Eshleman *et al* 1998, Scanlon *et al* 2010, Eshleman *et al* 2013]. Synoptic spring baseflow sampling in each of the five PR subcatchments was conducted from 1992–1994 period and, after a long cessation, was restarted in 2007 and repeated annually through 2016. All water samples were analyzed for nitrate concentrations using ion chromatography. The spatial relationship between mean catchment-scale *δ*^15^N and mean spring baseflow nitrate concentrations at PR for the period of observation was explored using linear regression. At UBR, mean annual flow-weighted concentrations, estimated using a multi-parameter loading model [Eshleman *et al* 2013], were compared against one year lagged catchment-scale *δ*^15^N values at the UBR site using simple linear regression. The one year lagged values were used because there was an assumed lag between N available in a given growing season and subsequent flush in the following dormant season. Likewise, linear regression was used to test the relationship between spring baseflow nitrate concentrations and one year lagged catchment-scale *δ*^15^N values at the PR headwater catchments. The relationship between mean April nitrate concentrations at UBR catchment-scale *δ*^15^N was also evaluated. Linear interpolation between catchment-scale *δ*^15^N time periods (e.g., between 1992 and 1994) was used to provide an annual record of catchment-scale *δ*^15^N values that is directly comparable to stream records since tree rings were analyzed in 2–3 year increments. For the Paine Run sites, this allowed three additional spring baseflow concentrations to be compared against catchment-scale *δ*^15^N (i.e., 1993, 2007, and 2009) and 10 additional mean April and mean annual flow-weighted concentration values at Upper Big Run, thus allowing the complete variability of observed nitrate concentrations to be considered. The difference in the number of spring samples between UBR and PR was due to the hiatus in sampling at PR. The imputation procedure described above may result in the underestimation of the coefficient standard error by increasing the degrees of freedom, so we carried out a complimentary comparison of non- interpolated one year lagged catchment-scale *δ*^15^N values and stream nitrate concentrations to further test the robustness of the relationship.

Disturbance within 45 m of the center of individual plots was described using the Disturbance Index (DI, [Healey *et al* 2005] applied to Landsat 5, 7 and 8 (TM/ETM+/OLI) data. As such, disturbance was quantified in 8 to 12% of the area in each catchment. All available tier I surface reflectance and quality assurance quality control (QAQC) data for Landsat 5, 7, and 8 were extracted for each cored plot and downloaded using the Google Earth Engine. Using the provided QAQC information, surface reflectance observations collected under suboptimal conditions (cloudy, cloud shadow, snow and ice, etc) were removed from the data set. From the data that passed quality control, the six Landsat multispectral bands were reduced to three orthogonal indices of brightness (B), greenness (G), and wetness (W) through the tasseled cap transformation [DeVries *et al* 2016]. The Disturbance Index (DI) is a simple linear combination of these indices (DI = B-(G + W)), where greener and wetter pixels indicate less disturbance and brighter, dryer, and browner pixels indicate greater disturbance. A continuous stable forest period (~5 to 15 years) for each individual plot was identified by referencing local logging maps provided by the Maryland State Forest Service and the North American Forest Dynamic data product, ‘Forest Disturbance History from Landsat, 1986–2010’ (supplemental figure 1). To standardize for sun-canopy-sensor geometry, we organized all ‘stable forest’ DI observations by day of year and used locally weighted regression models (i.e., LOESS) to model the average DI phenology. From the LOESS fit, expected DI for each day of year was estimated that account for canopy phenology and sun angle effects that reoccur each growing season. The difference between the observed and modeled DI values (ΔDI) was calculated for all observations, representing disturbance above (positive values) or below (negative values) the mean DI during the stable period for any given day of year. A mean growing season (May 1st to September 30th) ΔDI value was calculated using all Landsat observations that fell within the years corresponding to the cut tree ring segments. Similar approaches using various empirical models to describe Landsat phenology in forests have been applied elsewhere (e.g., [Zhu *et al* 2012, Elmore *et al* 2016] with the overall goal being to normalize for intra-annual variation so that inter-annual variation can be quantified. It should be clarified that mean catchment ΔDI was not quantified at the catchment scale (i.e., sampling all pixels within a watershed) and related to stream nitrate concentrations. This relationship has already been demonstrated in multiple studies [Townsend *et al* 2004, McNeil *et al* 2007, Eshleman *et al* 2009, Townsend *et al* 2012]. The primary motivation for this analysis was to ascertain the effect of ΔDI on wood *δ*^15^N.

Annual wet deposition of inorganic N (IN) and sulfur (S) along with annual temperature and precipitation for UBR and the PR sub-catchments were extracted for the locations of individual trees. Data sets used included wet deposition annual gradient maps published by the National Atmospheric Deposition Program for the 1980–2015 period and climate data published by the PRISM Climate group [Latysh and Wetherbee 2012]. Annual temperature values were determined by averaging extracted monthly temperatures from PRISM climatic maps. Little to any intra-site variation at UBR and PR in the climatic and deposition variables was observed due to the coarse spatial resolution (~4 km), so only the site averages were reported. Annual values of temperature, precipitation, and wet deposition of N and S deposition corresponding with the cut tree ring segments were averaged by taking the simple arithmetic mean and used in later statistical analyses to predict the inter-annual variation in tree-ring *δ*^15^N.

Annual inorganic S and N wet deposition, ΔDI, mean annual temperature, and mean annual precipitation were used as predictor variables to explain the inter-annual variation in tree ring *δ*^15^N. To avoid multicollinearity and to gain greater confidence in causal relationships, the linear effect of time was assessed first, and then removed from both predictor and response variables associated with each individual tree. Thus, the residuals from the suite of predictor variables were leveraged to explain detrended tree-ring *δ*^15^N residuals using multiple linear regression in SigmaPlot 14.0. Multicollinearity among the predictor variables was assessed using variance inflation factor (VIF, [Graham 2003]).

## Results

**Figure 2.**
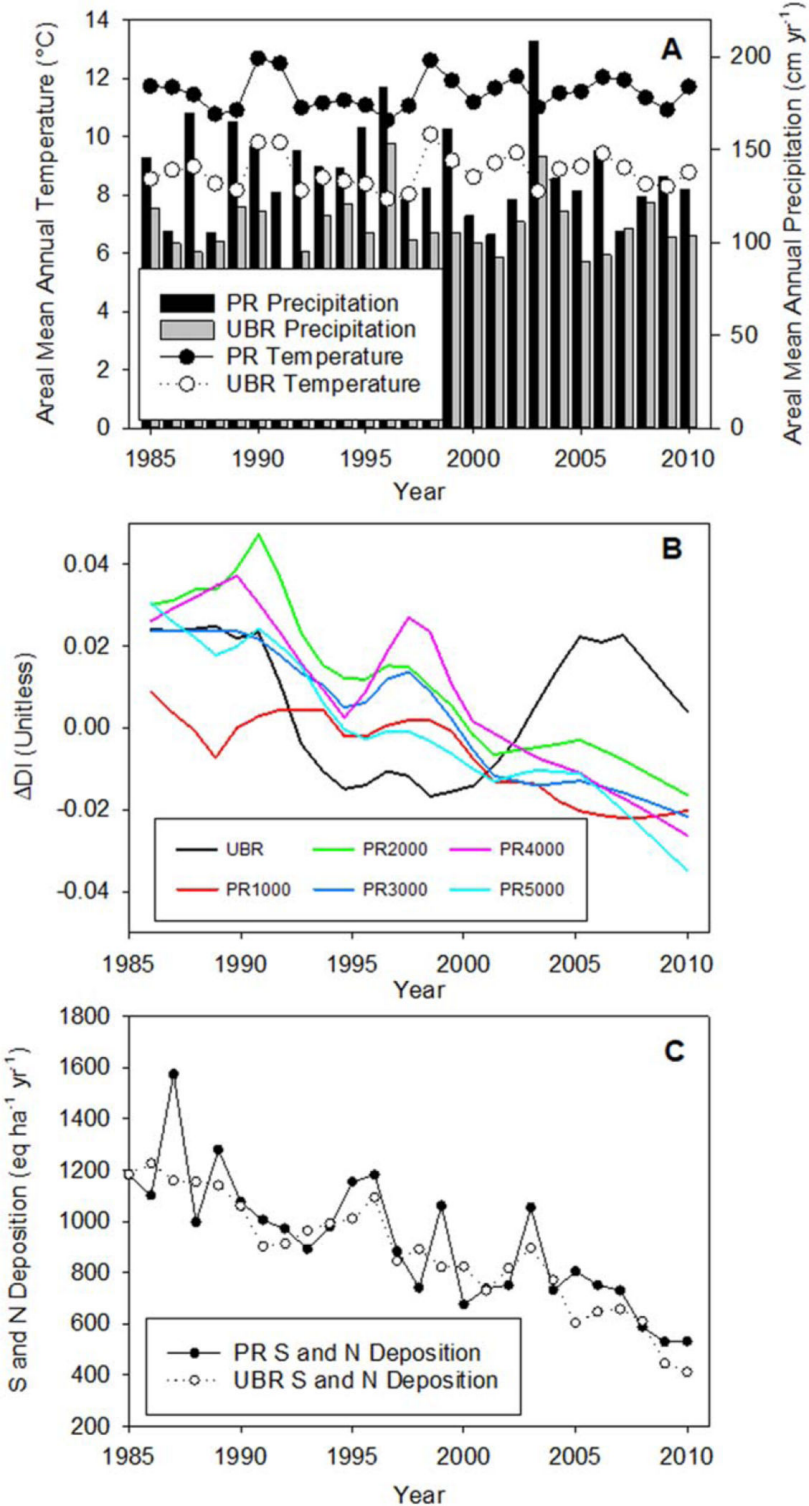
Time series of (A) annual temperature, precipitation, (B) mean disturbance index (ΔDI) for the population of sampled trees in individual catchments, and (C) annual S and N deposition at Paine Run (PR) and Upper Big Run (UBR). For (B) only, the LOESS functions fit to all ΔDI observations that corresponded to individual tree ring segments within a catchment are shown (raw ΔDI data illustrated in supplemental figures 2–7).

**Figure 3.**
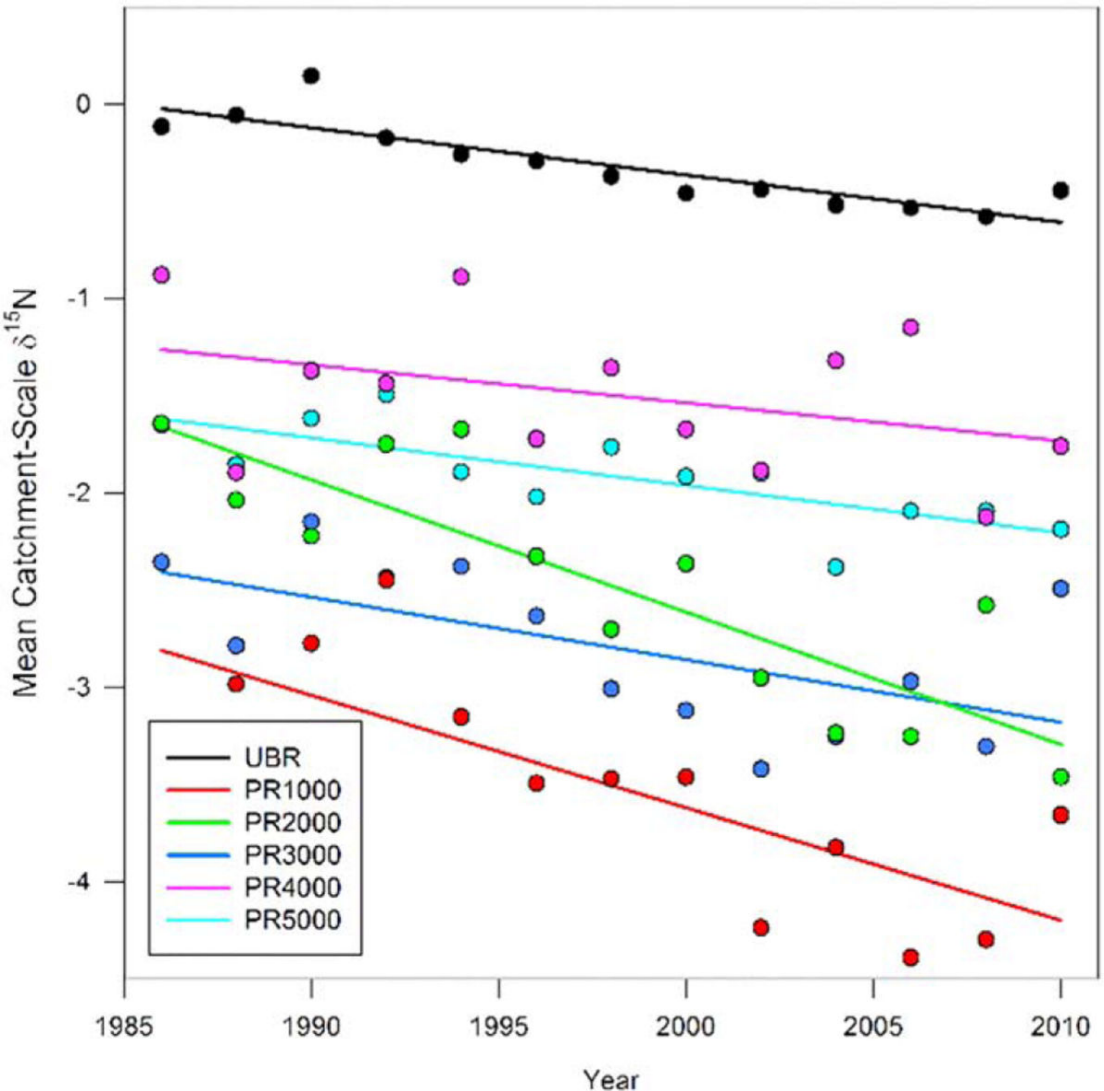
Time series of catchment-scale *δ*^15^Nfor Upper Big Run (UBR) and the five Paine Run (PR) sub-catchments and associated regression lines. Simple linear regression results indicated significant declines in catchment-scale *δ*^15^N for all catchments except for PR4000(UBR: y = −0.017 + 33.86, R^2^ = 0.65, p < 0.001; PR1000: y = −0.056 + 107.86, R^2^ = 0.68, p < 0.001; PR2000: y = −0.055 + 107.57, R^2^ = 0.70, p < 0.001; PR3000: y = −0.032 + 60.48, R^2^ = 0.47, p=0.003; PR4000: y = 0.013x−26.65, R^2^ = 0.06, p = 0.35; PR5000: y = −0.023 + 43.81, R2 = 0.60, p < 0.001). Raw tree-ring *δ*^15^ N time series illustrated in the supplemental (supplemental figures 2–7).

No long-term trends in annual precipitation or temperature were observed at our study catchments over the 25- year analysis period (figure 2(A)). LOESS curves of the annual change in disturbance index (ΔDI) of individual tree time series at UBR and PR suggested a period of disturbance from the mid-1980s to early 1990s for the population of trees (figure 2(B), supplemental figures 2–7). ΔDI generally declined after the late 1980s and early 1990s at all PR headwater catchments, which is consistent with remote sensing based disturbance classification products (using different methodologies) detecting disturbance in the late 1980s and early 1990s (figure 2(B), supplemental figure 1). ΔDI at UBR declined throughout the 1990s following logging then increased after 2000, coincident with another round of logging activities and reported incidents of gypsy moth defoliations (figure 2(B), supplemental figure 1; [Townsend *et al* 2012]). S and N deposition declined nearly 50% throughout the period of record at both sites (figure 2(C)). During this period, catchment-scale *δ*^15^N significantly decreased (p < 0.01) at all headwater catchments at PR and UBR throughout the period of record except for PR4000 (figure 3, supplemental table 1). Consistent with these aggregated values, 80 of the 96 individual trees showed linear declines in tree-ring *δ*^15^N of which twenty were significant (p < 0.05, supplemental tables 4, 5). Only 11 of the remaining 16 trees with positive linear increases in tree ring *δ*^15^N were significant (p < 0.05).

**Figure 4.**
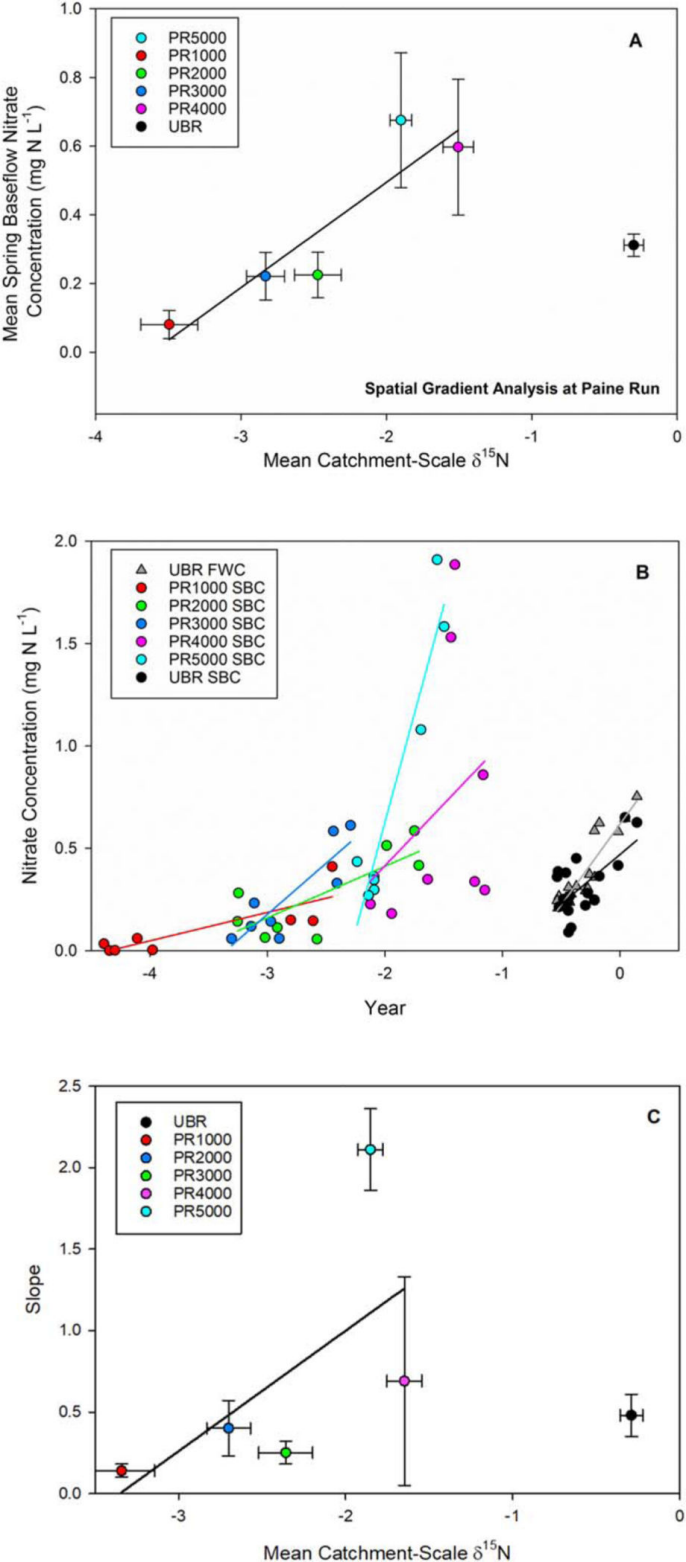
(A) Relationship between mean catchment-scale *δ*^15^N and spring baseflow nitrate concentrations across space over the period of record among the Paine Run (PR) and Upper Big Run (UBG) headwater catchments (y = 0.35x + 1.29, R^2^ = 0.86, p = 0.023 for data from PR). The error bars illustrate the standard error of the mean. (B) Relationships between 1-year lagged catchment scale *δ*^15^N and nitrate concentrations at individual PR headwater catchments and UBR; all regressions were significant except for PR4000 (UBR FWC: y = 0.79x + 0.62, R^2^ = 0.85, p < 0.01; UBR SBC (mean April):y = 0.48x + 0.47, R^2^ = 0.40, p < 0.01; PR1000: y = 0.14x + 0.55, R^2^ = 0.67, p < 0.01; PR2000: y = 0.25x + 0.53, R^2^ = 0.67, p < 0.01; PR3000: y = 0.40x + 1.05, R^2^ = 0.45, p = 0.04; PR4000: y = 0.69x + 0.94, R^2^ = 0.14, p < 0.32; PR5000: y = 2.11x + 4.23, R^2^ = 0.91, p < 0.01). (C) The linear relationship between mean catchment-scale *δ*^15^N and slope estimates based on the relationship between 1-year lagged catchment scale *δ*^15^N and nitrate concentrations for the PR catchments (y = 0.74x + 2.48, R^2^ = 0.19, p = 0.17). The data point from UBR was not included in the regression.

Across basins and over time at individual basins, catchment mean wood *δ*^15^N values were observed to have positive linear relationships with stream nitrate concentrations. Across 5 sub-basins at PR, mean spring baseflow nitrate concentrations (averaged across all years) increased linearly with increasing catchment mean wood *δ*^15^N (R^2^ = 0.86; P = 0.023, table 1). The slope of this relationship equates to a gradient in spring base flow nitrate concentration from 0.09 to 0.73 mg N l^−1^ being associated with a gradient in catchment mean wood *δ*^15^N from −3.6 to −1.5‰ (figure 4(A)). The portion of variance in nitrate concentration explained by the spatial gradient in mean wood *δ*^15^N observed at PR was removed by taking the difference between the mean predicted stream nitrate concentration from the observed nitrate concentration value in a given year. Temporal variation in wood *δ*^15^N was a significant model effect on nitrate concentrations (figure 4(B)), with a positive effect of catchment wood *δ*^15^N observed through time at four of the five Paine Run catchments (table 2). The results from this analysis were consistent with the coefficient estimates and standard error using the non-interpolated catchment- scale *δ*^15^N data (table 2). While the variance in stream nitrate concentrations explained was generally similar, the relationships were only significant in two of the four Paine Run catchments using the non-interpolated catchment-scale *δ*^15^N time series (table 2). Estimates of slope coefficients generally increased with increasing mean wood *δ*^15^N (p = 0.17, figure 4(C)). This positive correlation suggests that stream nitrate concentrations were least sensitive to annual variation in wood *δ*^15^N at sites with low wood *δ*^15^N (e.g., PR1000) and were most sensitive at sites with high wood *δ*^15^N at Paine Run (e.g., PR5000, figure 4(C)). Like the findings from PR, mean annual flow-weighted nitrate and spring baseflow nitrate concentrations at UBR were positively correlated with catchment wood *δ*^15^N (R^2^ = 0.88; P < 0.0001 and R^2^ = 0.43; P = 0.0017, respectively figure 4(B)); and these relationships were robust when using the non-interpolated catchment-scale *δ*^15^N time series with equivalent, significant slope estimates (table 2).

**Figure 5.**
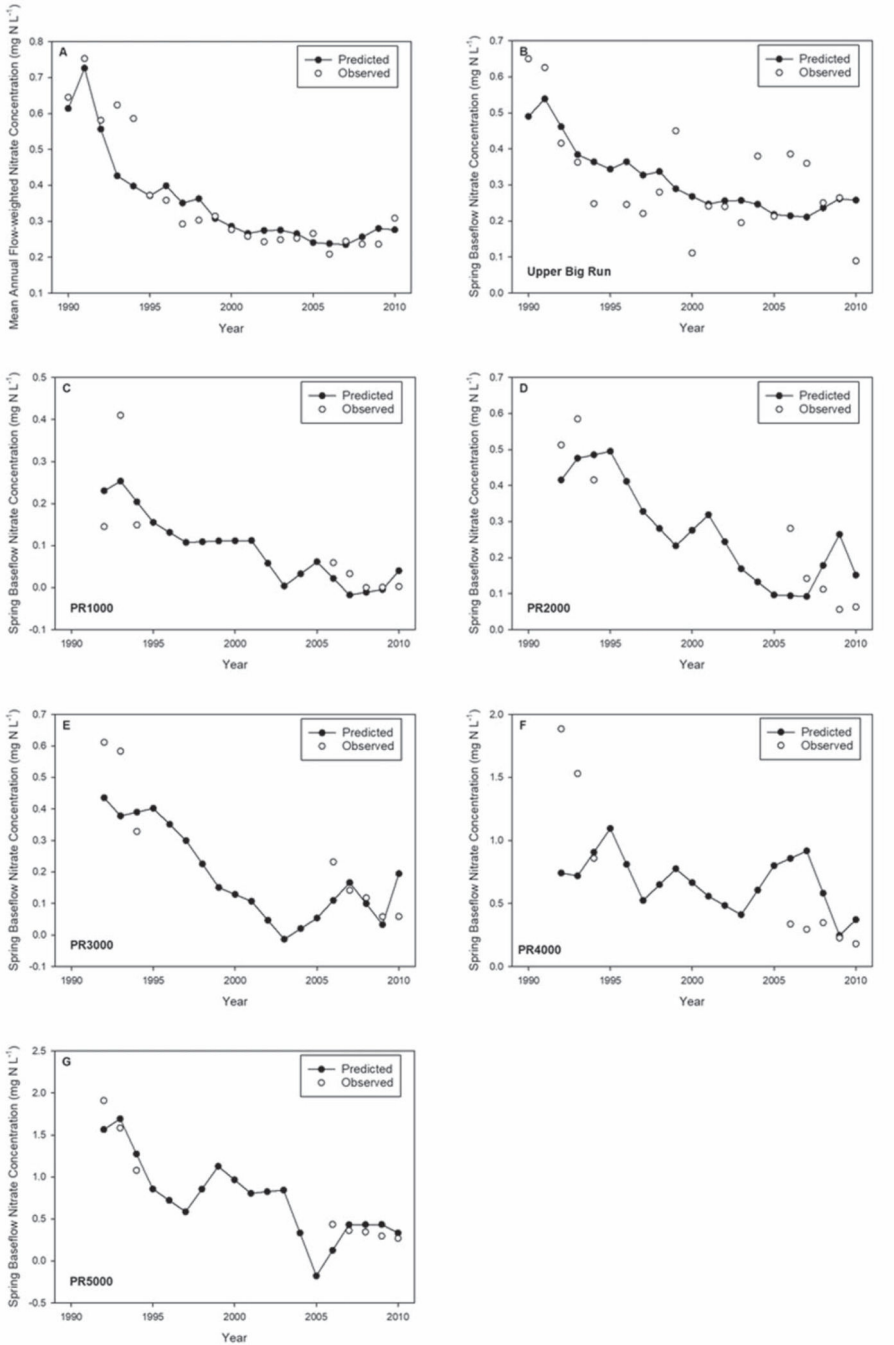
Predicted and observed mean annual flow-weighted nitrate concentrations at Upper Big Run (UBR) (panel A) along with predicted and observed spring baseflow nitrate concentrations for the five Paine Run (PR) headwater catchments and UBR (panel B-G).

Using the simple linear regression models (table 2), catchment-scale *δ*^15^N was used to model the temporal variability in spring baseflow and mean annual flow-weighted nitrate concentrations from 1990 to 2010 for UBR and 1992 to 2010 for PR. The 1-year lagged catchment-scale *δ*^15^N regression models were effective in capturing peak nitrate concentrations in the early 1990s followed by a decline to the 2000s in five of the six catchments (figure 5). Furthermore, the models were successful in generating the generally stable stream nitrate concentrations at UBR during the 2000s. Predicted nitrate concentration time series were generally smoother than the observed spring baseflow nitrate concentration time series (one sample per year), likely reflecting the interpolation of wood *δ*^15^N values between the measured 2 to 3-year increments, wood *δ*^15^N values being minimally affected by annual discharge, and the fact the N has the potential to translocate between adjacent tree rings.

After removing the linear effect of time, all predictor variables except for temperature and precipitation were significant in explaining the inter-annual variation in residual tree ring *δ*^15^N (table 3). Residual tree-ring *δ*^15^N decreased with increased S and N deposition (p < 0.001) and *Δ*DI residuals (p = 0.016). As such, higher rates of disturbance and acidic deposition decreased tree-ring *δ*^15^N. Among the significant predictor variables and after accounting for trends over time, S and N deposition was the most influential with the highest sum of squares, whereas *Δ*DI had the lowest sum of squares (table 3). The multiple linear regression model, though significant (p < 0.001), explained very little of the inter-annual variation of detrended tree-ring *δ*^15^N (R^2^ = 0.041) which could be partly due to the analytical precision of tree-ring *δ*^15^N. If using non-detrended data in a generalized linear model with a categorical variable of a tree identification code (i.e., the factor), the suite of predictor variables plus time explained upwards of 90% of the variance with similar slope coefficient estimates for S and N deposition as predicted by the multiple linear regression model (supplemental table 3), but multicollinearity amongst the predictor variables was identified (VIF > 14 for year and S and N deposition).

## Discussion

Our results strongly show that spatiotemporal variability in stream nitrate concentrations is correlated with a widely-used proxy for terrestrial N availability: wood *δ*^15^N. Positive relationships between catchment-scale *δ*^15^N and stream nitrate concentrations are consistent with the understanding that wood, foliar, and soil *δ*^15^N records are indicators of terrestrial N availability [Craine *et al* 2009, Gerhart and McLauchlan 2014]. Across space at the PR headwater catchments, those catchments with higher mean wood *δ*^15^N exhibited higher stream nitrate concentrations. This result suggests that catchments with more positive wood *δ*^15^N have higher terrestrial N availability (i.e., N supply greater than plant demand) and greater nitrate export. A similar observation was made in a watershed study comparing mean annual flow-weighted nitrate concentrations and non-normalized catchment-scale *δ*^15^N values in the Adirondacks, but that result was not considered conclusive since one of the predominant species in that study was not sampled because it was diseased [Sabo *et al* 2016a]. Moving beyond and consistent with dendroisotopic records, forested catchments with corresponding lower foliar and soil C:N ratios also typically have higher stream nitrate concentrations throughout the Northeast [Aber *et al* 1998, Aber *et al* 1989]. Further expanding this spatial analysis to include other catchments with stream nitrate concentrations and dendroisotopic records is needed to see if broader positive spatial relationships between stream and terrestrial N availability emerge despite differences in the underlying geology, forest management strategies, species composition, successional status, and topography among forest (akin to global foliar [N] and *δ*^15^N gradient relationships, Craine *et al* 2009).

Over time, periods with higher wood *δ*^15^N were associated with periods of higher stream nitrate concentrations regardless of the stream metric used (spring baseflow, annual flow-weighted, or mean April concentration). Catchment-scale *δ*^15^N regression models also successfully described the decadal stability of mean annual flow-weighted nitrate concentrations at UBR and were generally predictive of spring baseflow nitrate concentrations in five of the six catchments. These results indicate that a variety of nitrate concentration records may be used to compare against future records of wood *δ*^15^N. Similar to our results, a recent study found that wood *δ*^15^N records from a single species, tulip poplar (*Liriodendron tulipifera*), could explain inter-annual variation of stream nitrate concentrations at a catchment in Fernow Experimental Forest [Burnham *et al* 2016]. Other observational studies have suggested similar relationships through time [McLauchlan *et al* 2007, Sabo *et al* 2016a]. In combination, our results and previous studies suggest that wood *δ*^15^N models can be used to fill in temporal gaps in, and potentially extend, water quality records back in time. Furthermore, the positive correlation between wood *δ*^15^N and stream nitrate concentrations, a widely held index of terrestrial N availability [Vitousek and Melillo 1979, Aber *et al* 1989], provide useful information in the ongoing debate about the usefulness and interpretability of *δ*^15^N signatures in plant tissue [Craine *et al* 2018, Craine *et al* 2019, Hiltbrunner *et al* 2019]. While some argue that the *δ*^15^N signatures in plant tissue cannot be easily leveraged as a proxy for terrestrial N availability relative to plant demand due to the complexities associated with accounting for a plant’s unique physiological aspects interacting the with the source, transport, and isotopic fractionation of nitrogen [Tomlinson *et al* 2016, Burnham *et al* 2019, Hiltbrunner *et al* 2019], the positive relationship between stream nitrate concentrations and wood *δ*^15^N provide robust evidence indeed that *δ*^15^N signatures in plant tissue record terrestrial N availability [Craine *et al* 2009, Elmore *et al* 2016, Craine *et al* 2018].

At our sites, a positive correlation between the sensitivity of stream nitrate concentrations to wood *δ*^15^N was observed. Lower significant slope coefficient estimates (slope < 0.4) corresponded to catchments with lower mean catchment-scale *δ*^15^N (< −2.5‰) and nitrate concentrations over the period of record, suggesting the sensitivity of stream nitrate concentrations to changes in catchment-scale *δ*^15^N through time is partly a function of the amount of N available relative to plant demand at a site. This speculation is consistent with evidence that forested catchments that retain minimal amounts of atmospherically deposited N (i.e., the most N rich sites) have the greatest absolute reduction in stream nitrate yields and flow-weighted concentrations following declines in atmospheric N deposition [Eshleman *et al* 2013]. As such, forested areas with higher N availability relative to plant demand will demonstrate a greater per unit decline in nitrate concentrations as wood *δ*^15^N declines, evaluated on an annual basis. Whether this linear relationship holds outside the range of *δ*^15^N values observed at these catchments (−2.5 to 0‰), reaches an inflection point (i.e., a phase shift), or is a non-linear continuous response should be pursued in future research [Aber *et al* 1989, Lovett and Goodale 2011]. Further investigation into the nature of the terrestrial availability and nitrate concentration relationship is important to determine the trajectory of forest recovery from historically high rates of atmospheric N and S deposition.

Throughout the period of record, catchment-scale *δ*^15^N suggests that terrestrial N availability relative to plant demand generally declined through time, but trends at the scale of an individual tree were more variable. These clear declines in terrestrial N availability at the scale of the catchment and associated variability in individual tree-ring *δ*^15^N trends are consistent with other dendroisotopic studies reporting over a similar period throughout the eastern United States [Burnham *et al* 2016, Elmore *et al* 2016, Sabo *et al* 2016a, Mathias and Thomas 2018]. While species specific sensitivities to atmospheric N and S deposition [Horn *et al* 2018], soil acidification status [Sabo *et al* 2016a], and disturbance [Howard and McLauchlan 2015] may modify the trajectory of N availability, it is clear that N availability for forests in general has declined. This decline in terrestrial N availability, described by some as ‘oligotrophication’ [Elmore *et al* 2016, McLauchlan *et al* 2017, Groffman *et al* 2018], may contribute to future declines in forest productivity, especially as atmospheric N deposition continues to decline and CO_2_ concentrations continue to rise [Eshleman *et al* 2013, Lloret and Valiela 2016, Wang *et al* 2017, Craine *et al* 2018]. Although it is uncertain what the ultimate impacts of declining N availability will be on the terrestrial compartment, it is clear that less terrestrial N is reaching the already oligotrophic streams of Upper Big Run and Paine Run [Dodds *et al* 1998].

Our water quality analysis demonstrated that stream nitrate concentrations are largely driven by changes in terrestrial N availability, but what are the drivers of longer-term trends and the general inter-annual variation in terrestrial N availability? Recent studies have applied a suite of statistical approaches to explain *δ*^15^N through time [Elmore *et al* 2016, Mathias and Thomas 2018], but collinearity issues among potential predictor variables and time make it difficult to statistically attribute the influence of various environmental drivers on wood *δ*^15^N. We applied a conservative approach in which the linear correlation with time was removed from both the response and predictor variables to (1) better identify causal relationships between predictor and response variables by comparing unique information in the time series and (2) gain greater confidence in the sign and error of coefficient estimates by eliminating multicollinearity. Our resulting regression model was significant, but only explained a small portion (4%) of the detrended residual *δ*^15^N of the 96 trees. This low explanatory power speaks to the complexity of statistically modeling individual tree-ring *δ*^15^N responses using spatially coarse environmental datasets, particularly when trends over time are evaluated separately and removed from the time series. Furthermore, our decision to sample wood in 2–3 year segments rather than annual increments reduced our ability to explain variation at higher temporal frequencies. Changes in the *δ*^15^N signature of atmospheric N deposition was also not considered in this analysis since such data are not available. Some have argued that the impacts of the *δ*^15^N signature in atmospheric N deposition have minimal influence on plant tissue *δ*^15^N [McLauchlan *et al* 2017, Craine *et al* 2018], although *δ*^15^N of deposition is potentially influential in certain locales with strong emission sources [Saurer *et al* 2004]. Leveraging sub-annual datasets like identifying maximum disturbance in a given year [Townsend *et al* 2004] or spring timing [Elmore *et al* 2016] or collapsing the variance of the detrended residual *δ*^15^N into a catchment-scale *δ*^15^N residual, might improve the explanatory power, but generally would require data with higher temporal frequency or spatial density.

Despite the aforementioned limitations, the significant coefficient estimates offer some insights into forest- wide drivers of terrestrial N availability and parallel observations from other studies using different methods to track changes in terrestrial N and carbon cycling After removing the long-term trend of declining S and N deposition and declining wood *δ*^15^N, S and N deposition exhibited a negative relationship with wood *δ*^15^N (table 3), suggesting that lower rates of acidic deposition are associated with enhanced N availability on inter- annual scales. It has been observed in the lab and in the field that decreased soil pH and aluminum mobilization can lead to increased retention of soil organic matter and shifts in microbial activities, but increases in pH can lead to increased loss of organic matter and nitrogen [Sinsabaugh *et al* 2004, Sinsabaugh 2010, Oulehle *et al* 2011, Oulehle *et al* 2017, Lawrence *et al* 2019]. The predominantly deciduous forests of UBR and PR lie on poorly buffered, infertile soils and are likely experiencing deacidification as the soils regain base cations and limit toxic aluminum mobilization after decades of elevated rates of acidic deposition [Lawrence *et al* 2015, Kline *et al* 2016, Groffman *et al* 2018]. Studies that have observed deacidification suggest that organic pools of carbon and N are now being mineralized and lost to streams at both experimentally manipulated and reference catchments [Oulehle *et al* 2011, Johnson *et al* 2014, Rosi-Marshall *et al* 2016, Oulehle *et al* 2017, Lawrence *et al* 2019]. As soils continue to recover from past acidic deposition, increased ammonification/nitrification and subsequent denitrification, an emission pathway that greatly enriches soil ^15^N [Kendall and McDonnell 2012], may be a positive offset to the decline in wood *δ*^15^N and terrestrial N availability. Ultimately, the magnitude and duration of this release of legacy N and its impact on terrestrial N availability and stream nitrate loss is likely conditional on the proportion of unprocessed atmospheric nitrate reaching streams [Sabo *et al* 2016b] and forest growth responses to increased base cation availability [Battles *et al* 2013], longer growing seasons [Elmore *et al* 2016], increased CO_2_ [Norby *et al* 2016], and improved air quality [Mathias and Thomas 2018].

Wood *δ*^15^N also declined in response to disturbance, suggesting that ephemeral disturbances [McNeil *et al* 2007], consisting mainly of defoliation events and selective silvicultural activities, act to reduce N availability in these systems over the long-term. While it is apparent that short-lived pulses of nitrate loss to streams are often observed immediately after a disturbance [Eshleman 2000], the loss of this nitrate via increased denitrification may be insufficient to increase the isotopic signature of plant available N pools in the soils and it is unclear if this pulse of N is even available to defoliated plants. Furthermore, the enhancement of a long-term growth sink ultimately reduces N availability in the ecosystem. This finding is consistent with a study carried out in UBR and the surrounding Savage River State Forest which reported that the N content of leaves was lower at sites that had experienced disturbance relative to undisturbed sites [McNeil *et al* 2007]. Similar conclusions have also been reported based on soil *δ*^15^N data from forests of the Pacific Northwest [Perakis *et al* 2015]. An alternative, though less likely, explanation to the effects of disturbance of wood *δ*^15^N and terrestrial N availability described above is that increased nitrification rates spurred by decreased vegetative uptake may temporarily reduce *δ*^15^N signature of nitrate in the soil. This decline in the isotopic signature of plant available nitrogen in the soil is a transient situation where the system can have a simultaneous increase in N availability yet have a reduction in the *δ*^15^N signature of plant available nitrate. The interplay of short and long-term impacts of disturbance on *δ*^15^N in soils and plants and more importantly on terrestrial N availability in ecosystems should be further explored.

Declines in terrestrial N availability over the past 30 years drove reductions in stream nitrate concentration and loss at our study sites, consistent with reported declines in nitrate export from forested catchments throughout the mid-Atlantic and the Northeast over a similar time period [Kothawala *et al* 2011, Eshleman *et al* 2013]. The impacts of decreased nitrate loss to streams may help diminish the occurrence of episodic acidification in headwater systems and chronic eutrophication in downstream rivers, lakes, and estuaries—welcomed improvements to these chronically impaired aquatic ecosystems. Overall, it is likely that forest N availability has and will continue to impact downstream N availability, and that these trends can be monitored through dendroecological evaluation of wood *δ*^15^N.

## Supplementary Material

Sup. Material

## Figures and Tables

**Table 1.** Results of the linear regression analysis of mean catchment-scale wood *δ*^15^N as a predictor of mean spring baseflow nitrate concentrations over the period of record among the five Paine Run (PR) headwater catchments.

PR spatial model	Coefficients	Standard error	P-value	R^2^

Intercept	1.29	0.21	<0.01	0.86
Slope	0.35	0.08	0.02	

**Table 2.** Results of the linear regression analyses modeling the effect of 1-year lagged catchment scale wood *δ*^15^N on (1) observed mean annual flow-weighted and mean April nitrate concentrations at Upper Big Run (UBR), (2) spring baseflow nitrate concentrations at individual Paine Run (PR) headwater catchments after factoring out the influence of the spatial gradient and using the interpolated and non-interpolated wood *δ*^15^N time series.

	Coefficients		Standard Error		P-value		R^2^	
				
UBR Mean Annual Flow-Weighted Concentration	Interpolated	Non-Interpolated	Interpolated	Non-Interpolated	Interpolated	Non-Interpolated	Interpolated	Non-Interpolated

Intercept	0.62	0.613	0.03	0.0295	<0.01	<0.001	0.85	0.88
Slope	0.79	0.793	0.07	0.0843	<0.01	<0.0001		
UBR Mean April Concentration								
Intercept	0.47	0.48	0.05	0.0593	<0.01	<0.001	0.40	0.521
Slope	0.48	0.559	0.13	0.17	<0.01	0.008		
PR1000 Intercept	0.55	0.378	0.14	0.066	<0.01	0.011	0.67	0.843
Slope	0.14	0.0859	0.04	0.0181	<0.01	0.018		
PR2000								
Intercept	0.53	0.842	0.18	0.271	0.03	0.053	0.67	0.473
Slope	0.25	0.219	0.07	0.102	<0.01	0.122		
PR3000								
Intercept	1.05	1.507	0.46	0.515	0.06	0.061	0.45	0.548
Slope	0.40	0.446	0.17	0.184	0.04	0.094		
PR4000								
Intercept	0.94	1.984	1.01	1.76	0.38	0.341	0.14	0
Slope	0.69	0.855	0.64	1.17	0.32	0.518		
PR5000								
Intercept	4.23	5.014	0.50	0.931	<0.01	0.013	0.91	0.832
Slope	2.11	2.164	0.25	0.474	<0.01	0.02		

**Table 3.** Results of the multiple linear regression analysis assessing the effects of detrended annual S and N deposition, disturbance (ΔDI), annual precipitation, and annual temperature on detrended tree ring *δ*^15^N (n = 973).

	COEFFICIENT	STANDARD ERROR	SUM OF SQUARES	P	STANDARDIZED COEFFICIENT	VIF

INTERCEPT	0.000 236	0.0133		0.986		
S AND N DEPOSITION RESIDUAL	−0.001 33	0.000 220	5.920	<0.001	−0.19	1.027
ΔDI RESIDUAL	−0.994	0.405	1.170	0.014	−0.0793	1.051
PRECIPITATION RESIDUAL	0.001 38	0.001 05	0.231	0.189	0.045	1.158
TEMPERATURE RESIDUAL	0.0245	0.0349	0.0853	0.482	0.0237	1.147
REGRESSION				p < 0.001	R^2^ = 0.043	
