# Supplementary material for: Positive correlation between wood δ 15N and stream nitrate concentrations in two temperate deciduous forests
Source: Environ Res Commun. Author manuscript; Available in PMC 2022 Oct 27. (PMC9610404; doi:10.1088/2515-7620/ab77f8)
Supplement: Sup. Material [file NIHMS1579159-supplement-Sup__Material.pdf]

Supplementary Figures and Tables

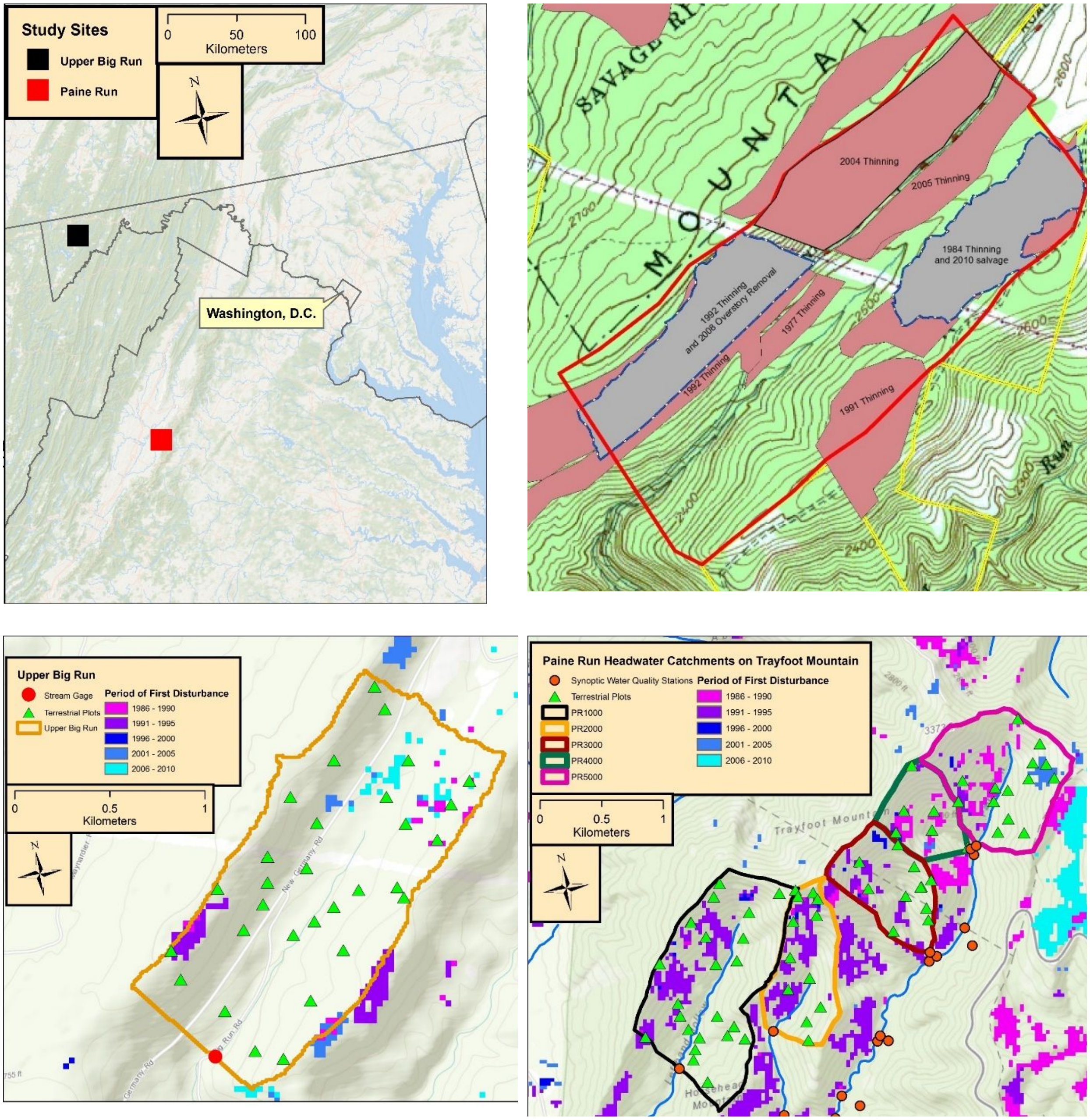

Supplemental Fig. 1. (A) Study site locations within the mid-Atlantic of the United States, (B) illustration of the areal extent of logging activities at Upper Big Run since 1970 (Overlapping red polygons represent more recent logging activities), (C, D) Maps of Upper Big Run and the five headwater catchments of Paine Run subjected to tree coring along with detected periods of disturbance recorded by the North American Forest Dynamic data product, "Forest Disturbance History from Landsat, 1986-2010". Logging map is courtesy of the Maryland State Forest Service.

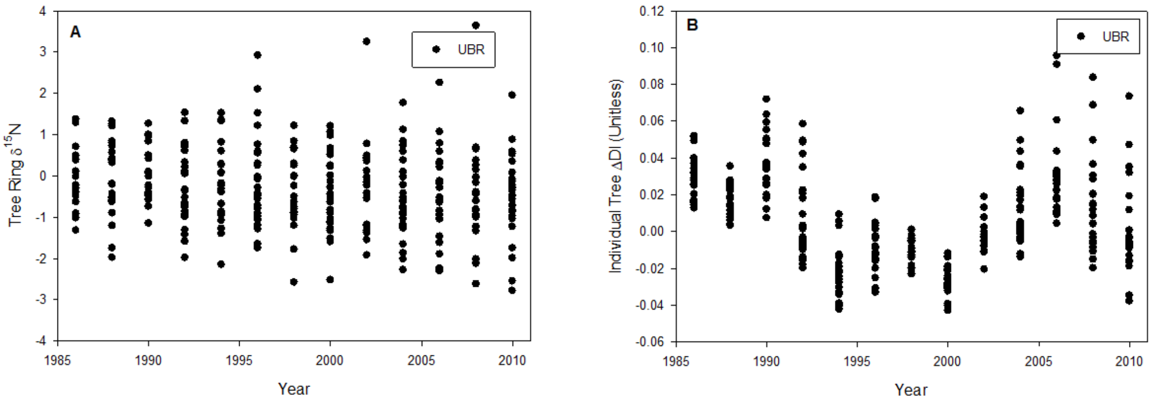

Supplemental Fig. 2. Mean ΔDI values corresponding to the tree-ring segments and tree-ring δ15N values at UBR .

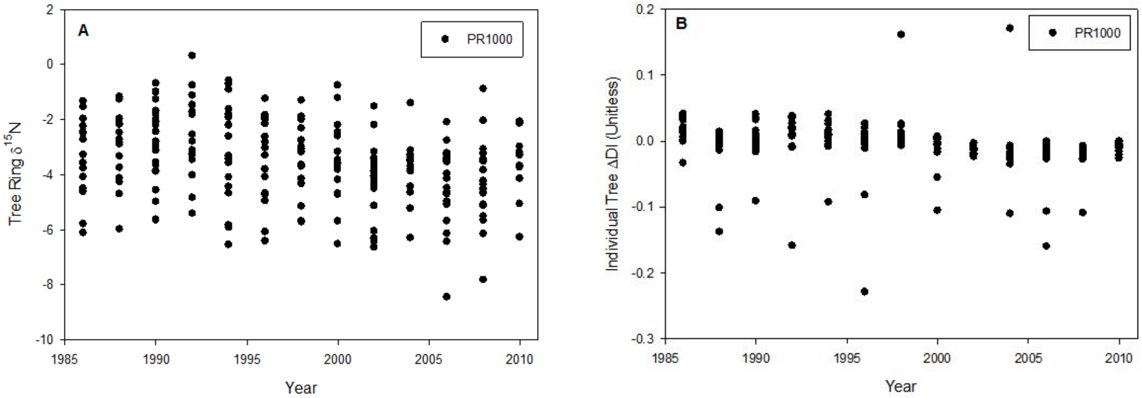

Supplemental Fig. 3. Mean ΔDI values corresponding to the tree-ring segments and tree-ring $\delta^{15}N$ values at PR1000.

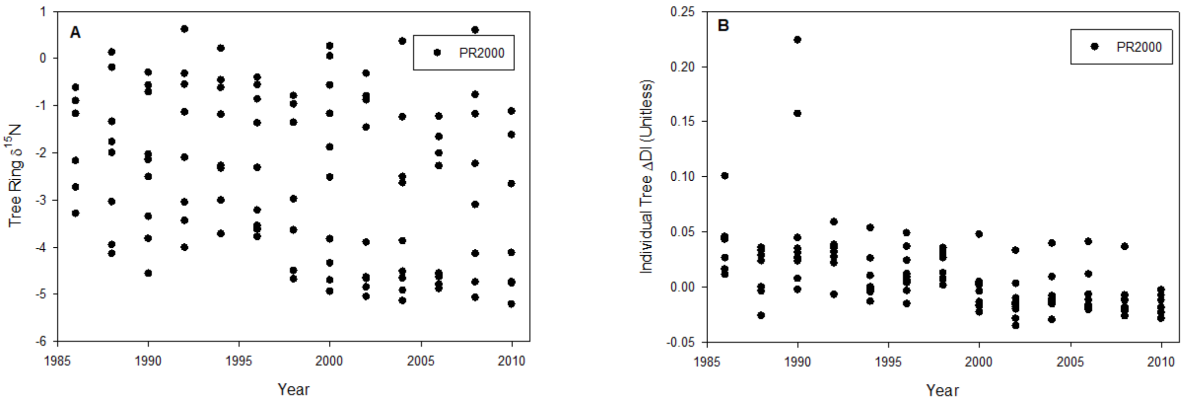

Supplemental Fig. 4. Mean ΔDI values corresponding to the tree-ring segments and tree-ring $\delta^{15}N$ values at PR2000.

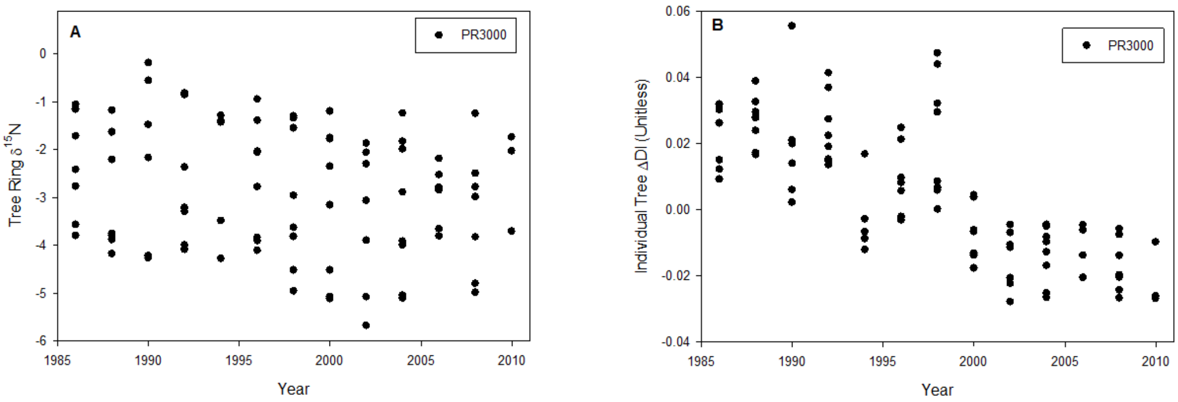

Supplemental Fig. 5. Mean ΔDI values corresponding to the tree-ring segments and tree-ring $\delta^{15}N$ values at PR3000.

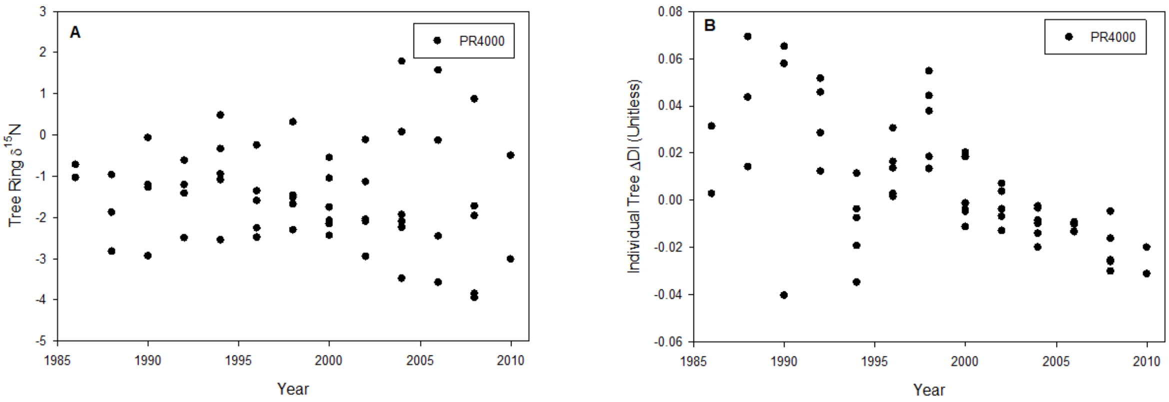

Supplemental Fig. 6. Mean ΔDI values corresponding to the tree-ring segments and tree-ring $\delta^{15}N$ values at PR4000.

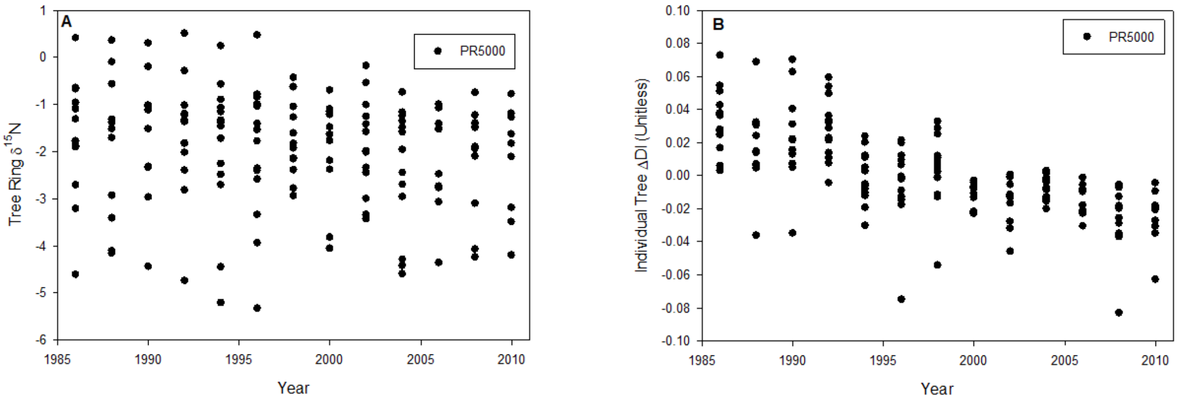

Supplemental Fig. 7. Mean ΔDI values corresponding to the tree-ring segments and tree-ring $\delta^{15}N$ values at PR5000.

Supplemental Table 1. Statistical output of the simple linear regression analysis of the relationship between time and catchment-scale $\delta^{15}N$.

		Coefficient	Std. Error	t	P	Model R^2
**UB**	Constant	33.859	6.716	5.042	<0.001	0.62
	Time	-0.0171	0.00337	-5.085	<0.001	
**PR1000**	Constant	107.856	19.372	5.568	<0.001	0.68
	Time	-0.0557	0.00971	-5.74	<0.001	
**PR2000**	Constant	107.568	19.395	5.546	<0.001	0.68
	Time	-0.0551	0.00972	-5.668	<0.001	
**PR3000**	Constant	60.475	16.723	3.616	0.003	0.47
	Time	-0.0317	0.00838	-3.778	0.002	
**PR4000**	Constant	-26.651	25.945	-1.027	0.322	0.0
	Time	0.0125	0.013	0.964	0.352	
**PR5000**	Constant	43.81	9.336	4.692	<0.001	0.60
	Time	-0.0229	0.00468	-4.891	<0.001	

Supplemental Table 2. Using the non-interpolated wood $\delta^{15}N$ dataset, results from the linear regression analyses modeling the effect of 1-year lagged catchment scale wood $\delta^{15}N$ on (1) observed mean annual flow-weighted and spring baseflow nitrate concentrations at UBR, (2) spring baseflow nitrate concentrations at individual PR headwater catchments after factoring out the influence of the spatial gradient.

**UBR Mean Annual Flow-Weighted Concentration**	*Coefficients*	*Standard Error*	*P-value*	R^2
Intercept	0.613	0.0295	<0.001	0.88
Slope	0.793	0.0843	<0.0001	
**UBR Spring Baseflow Concentration**				
Intercept	0.48	0.0593	<0.001	0.521
Slope	0.559	0.17	0.008	
**PR1000**				
Intercept	0.378	0.066	0.011	0.843
Slope	0.0859	0.0181	0.018	
**PR2000**				
Intercept	0.842	0.271	0.053	0.473
Slope	0.219	0.102	0.122	
**PR3000**				
Intercept	1.507	0.515	0.061	0.548
Slope	0.446	0.184	0.094	
**PR4000**				
Intercept	1.984	1.76	0.341	0
Slope	0.855	1.17	0.518	
**PR5000**				
Intercept	5.014	0.931	0.013	0.832
Slope	2.164	0.474	0.02	

Supplemental Table 3. Statistical output of the general linear model analysis of the relationship among time, precipitation, disturbance temperature, and S and N deposition vs. tree ring $\delta^{15}N$.

Analysis of Variance for the Equal Slopes Model:

**Source of Variation**	**DF**	**SS**	**MS**	**F**	**P**
Tree_ID	95	2107.007	22.179	68.010	<0.001
Year	1	25.639	25.639	78.619	<0.001
Precip	1	1.385	1.385	4.247	0.040
Disturb	1	0.536	0.536	1.644	0.200
S_N dep	1	6.138	6.138	18.823	<0.001
Residual	870	283.718	0.326	--	--
Total	969	3178.821	3.281	--	--
**R=0.954**		Rsq=0.911	Adj Rsq= 0.901		

Supplemental Table 4. Statistical output of the general linear model analysis of the relationship between time and tree-ring $\delta^{15}N$.

Analysis of Variance for the Interaction Model:

Source of Variation	DF	SS	MS	F	P
Tree_ID	95	115.741	1.218	5.459	<0.001
Year	1	58.27	58.27	261.088	<0.001
Tree_ID x Year	95	117.061	1.232	5.521	<0.001
Residual	781	174.304	0.223	--	--
Total	972	3191.185	3.283	--	--
R = 0.972		Rsqr = 0.945	Adj Rsqr = 0.932		

Supplemental Table 5 Slope coefficient outputs the general linear model analysis of the relationship between time and tree-ring $\delta^{15}N$

Tree_ID	**Scientific_Name**	Coefficient	95%Conf-L	95%Conf-U	P	Tree_ID		Coefficient	95%Conf-L	95%Conf-U	P
**BR26**	*Prunus serotina*	-0.0368	-0.0412	-0.0323	<0.01	**PR1023**	*Quercus prinus*	-0.02866	-0.0301	0.0464	0.676
**BR10I**	*Liriodendron tulipifera*	0.0331	0.0415	0.0984	<0.01	**PR1025**	*Quercus rubra*	-0.1294	-0.137	-0.0482	<0.01
**BR11II**	*Acer saccharum*	0.0026	-0.00636	0.0852	0.091	**PR1027**	*Quercus prinus*	-0.0654	-0.0729	0.0158	0.207
**BR12II**	*Quercus rubra*	-0.0773	-0.08	-0.00087	0.045	**PR1028**	*Quercus prinus*	-0.03514	-0.0336	0.0369	0.926
**BR13I**	*Quercus rubra*	-0.0825	-0.0843	-0.00705	0.021	**PR1031**	*Quercus prinus*	-0.0511	-0.0546	0.0261	0.488
**BR14I**	*Prunus serotina*	-0.0112	-0.0131	0.0643	0.194	**PR2001**	*Quercus prinus*	-0.05	-0.0604	0.034	0.583
**BR15I**	*Quercus rubra*	-0.0668	-0.0765	0.0166	0.207	**PR2003**	*Quercus rubra*	-0.0647	-0.0701	0.0142	0.193
**BR16II**	*Acer saccharum*	-0.03592	-0.0345	0.0363	0.961	**PR2004**	*Quercus prinus*	0.0318	0.0193	0.118	0.006
**BR17I**	*Acer saccharum*	0.0272	0.0128	0.115	0.014	**PR2005**	*Quercus rubra*	-0.0674	-0.0649	0.00369	0.08
**BR18I**	*Tsuga canadensis*	-0.03929	-0.0297	0.0247	0.857	**PR2006**	*Quercus prinus*	0.0294	0.0125	0.12	0.016
**BR19II**	*Quercus rubra*	-0.019	-0.0183	0.0538	0.334	**PR2008**	*Quercus prinus*	-0.0774	-0.0749	-0.00631	0.02
**BR20II**	*Acer saccharum*	-0.0234	-0.0281	0.0548	0.527	**PR2009**	*Quercus prinus*	-0.0593	-0.0705	0.0256	0.359
**BR21I**	*Acer rubrum*	-0.0159	-0.0225	0.0642	0.345	**PR2010**	*Quercus prinus*	-0.0701	-0.0772	0.0106	0.137
**BR22II**	*Acer rubrum*	-0.0059	-0.023	0.0849	0.261	**PR2011**	*Quercus rubra*	-0.0088	-0.0107	0.0668	0.156
**BR23II**	*Acer saccharum*	-0.0018	-0.00185	0.0719	0.063	**PR2012**	*Quercus rubra*	-0.0327	-0.0305	0.0388	0.816
**BR24II**	*Quercus prinus*	-0.03195	-0.0307	0.0404	0.789	**PR2013**	*Quercus prinus*	-0.0885	-0.0954	-0.00805	0.02
**BR25II**	*Quercus rubra*	-0.0064	-0.0181	0.0789	0.219	**PR5013**	*Liriodendron tulipifera*	0.0579	0.0447	0.145	<0.001
**BR27I**	*Acer saccharum*	-0.03131	-0.0351	0.046	0.79	**PR3003**	*Quercus prinus*	-0.03742	-0.0451	0.0439	0.978
**BR28II**	*Tsuga canadensis*	-0.03582	-0.0372	0.0392	0.96	**PR3004**	*Quercus prinus*	0.0062	-0.011	0.0969	0.118
**BR29II**	*Quercus alba*	-0.03889	-0.0448	0.0406	0.923	**PR3005**	*Quercus prinus*	-0.0618	-0.0593	0.00935	0.154
**BR2I**	*Acer saccharum*	-0.0044	0.00165	0.0632	0.039	**PR3006**	*Quercus prinus*	-0.0697	-0.0816	0.0157	0.185
**BR30I**	*Quercus rubra*	-0.0732	-0.0781	0.00538	0.088	**PR3007**	*Quercus prinus*	-0.0862	-0.0938	-0.00511	0.029
**BR31I**	*Acer rubrum*	-0.03117	-0.0322	0.0435	0.77	**PR3008**	*Quercus prinus*	-0.03292	-0.0372	0.045	0.853
**BR3I**	*Quercus rubra*	-0.03566	-0.0326	0.0349	0.947	**PR3009**	*Quercus prinus*	-0.03539	-0.0544	0.0572	0.961
**BR4II**	*Tsuga canadensis*	0.0032	-0.00085	0.0809	0.055	**PR3010**	*Pinus rigida*	0.0101	0.00901	0.0848	0.015
**BR5I**	*Acer saccharum*	0.0267	0.0245	0.102	0.001	**PR3011**	*Quercus rubra*	-0.0874	-0.0849	-0.0163	0.004
**BR6II**	*Acer saccharum*	-0.03848	-0.0457	0.0423	0.94	**PR3012**	*Pinus rigida*	-0.0337	-0.0757	0.0819	0.939
**BR7I**	*Tsuga canadensis*	-0.0595	-0.058	0.0125	0.206	**PR1019**	*Pinus strobus*	-0.1111	-0.113	-0.0356	<0.001
**BR8I**	*Tsuga canadensis*	-0.0436	-0.0371	0.0235	0.659	**PR4001**	*Carya glabra*	-0.02868	-0.0458	0.0621	0.768
**BR9I**	*Quercus rubra*	-0.1162	-0.128	-0.0309	0.001	**PR4002**	*Acer saccharum*	0.2852	0.24	0.404	<0.01
**PR1002**	*Quercus velutina*	-0.0726	-0.0769	0.00535	0.088	**PR4003**	*Quercus rubra*	-0.1131	-0.113	-0.0392	<0.01
**PR1003**	*Quercus rubra*	-0.1052	-0.105	-0.032	<0.01	**PR4006**	*Carya ovata*	-0.0808	-0.0918	0.0039	0.072
**PR1005**	*Quercus rubra*	-0.0213	-0.0311	0.0621	0.514	**PR4007**	*Quercus prinus*	0.0303	0.0254	0.109	0.002
**PR1006**	*Quercus prinus*	-0.0521	-0.0553	0.0248	0.455	**PR5001**	*Pinus strobus*	-0.1356	-0.132	-0.0655	<0.01
**PR1007**	*Quercus prinus*	-0.0841	-0.109	0.0141	0.131	**PR5002**	*Betula lenta*	-0.0053	-0.0185	0.0815	0.216
**PR1008**	*Pinus virginiana*	0.0142	0.0108	0.0912	0.013	**PR5004**	*Tilia americana*	-0.0137	-0.0141	0.0603	0.223
**PR1009**	*Pinus rigida*	-0.04099	-0.0518	0.0434	0.863	**PR5005**	*Carya glabra*	-0.066	-0.0683	0.00981	0.142
**PR1010**	*Quercus rubra*	-0.08	-0.0859	-0.00051	0.047	**PR4000**	*Acer rubrum*	-0.0515	-0.0578	0.0283	0.502
**PR1011**	*Quercus prinus*	-0.0839	-0.0876	-0.00668	0.022	**PR5007**	*Acer rubrum*	-0.04417	-0.0404	0.0257	0.662
**PR1012**	*Quercus rubra*	-0.1388	-0.14	-0.064	<0.01	**PR5008**	*Prunus serotina*	0.0037	-0.0111	0.0921	0.124
**PR1013**	*Quercus prinus*	-0.0612	-0.0683	0.0195	0.276	**PR5009**	*Acer rubrum*	-0.0089	-0.00798	0.0639	0.127
**PR1014**	*Quercus prinus*	-0.1121	-0.121	-0.0292	0.001	**PR5010**	*Acer rubrum*	-0.0082	-0.0154	0.0726	0.202
**PR1015**	*Quercus prinus*	0.0015	-0.00315	0.0797	0.07	**PR5011**	*Acer rubrum*	-0.0904	-0.0992	-0.00808	0.021
**PR1016**	*Quercus prinus*	-0.0226	-0.0263	0.0546	0.493	**PR5012**	*Quercus prinus*	0.0312	0.0284	0.108	<0.01
**PR1017**	*Quercus ilicifolia*	-0.052	-0.0879	0.0575	0.681	**PR5014**	*Quercus prinus*	-0.0228	-0.0207	0.0487	0.428
**PR1018**	*Pinus rigida*	-0.0711	-0.0799	0.0113	0.14	**PR5015**	*Liriodendron tulipifera*	-0.0143	-0.0118	0.0568	0.199
**PR1021**	*Quercus prinus*	-0.0999	-0.0987	-0.0276	<0.01	**PR5016**	*Acer rubrum*	-0.0579	-0.0666	0.0245	0.364
**PR1022**	*Quercus prinus*	-0.0748	-0.0861	0.01	0.121	**PR5017**	*Pinus strobus*	-0.018	-0.0155	0.0531	0.282
